# ﻿Mitochondrial DNA reveals two recent diverged lineages in *Amphioctopusaegina* (Gray, 1849) (Cephalopoda, Octopodidae) across the Leizhou Peninsula: a marine ecoregion barrier

**DOI:** 10.3897/zookeys.1179.96015

**Published:** 2023-09-14

**Authors:** Yantao Liu, Long Hou, Liqin Liu, Amna Sulaman, Faiz Muhammad

**Affiliations:** 1 National Engineering Research Centre of Marine Facilities Aquaculture, College of Marine Sciences and Technology, Zhejiang Ocean University, Zhoushan, China Zhejiang Ocean University Zhoushan China; 2 Center of Excellence in Marine Biology, University of Karachi, Karachi, Pakistan University of Karachi Karachi Pakistan

**Keywords:** *A.aegina*, genetic structure, marble octopus, mtDNA sequences, population divergence, sand-bird octopus

## Abstract

*Amphioctopusaegina* is an economically important species that has been intensively exploited in the marine areas along the Chinese coast. However, the genetic variation and population genetic structure, which would provide valuable information for their fisheries management, have rarely been investigated. In this study, the genetic variation within and among four *A.aegina* populations throughout its full distribution range were estimated based on mitochondrial cytochrome b DNA sequences. Our results indicated low (Qinzhou) to high (Dongshan) genetic diversities among the four populations. Analysis of molecular variance (AMOVA), ΦST statistics, phylogenetic tree and haplotype networks revealed two significant (*p* < 0.01) divergent lineages with a ΦST value of 0.7116 between them, one from a population in Qinzhou and the other from the remaining three populations of Dongshan, Huizhou and Zhanjiang. However, the low genetic distance (0.0032) and only two fixed substitutions between them suggest their recent divergence is possibly due to the last glacial period barriers to gene flow produced by the Leizhou Peninsula. The observed lineage divergence suggests that populations of *A.aegina* in China are genetically subdivided and may represent evolutionary lineages that should be managed individually.

## ﻿Introduction

Understanding the population genetic structure of a target fishery species is an important component of successful and sustainable management of fishery resources (Garoia et al. 2007). Determination of the population genetic structure can provide essential information regarding the stock composition, evolutionary history and population dynamics, which are all useful to underpin scientific management and sound conservation practices for fishery resources, and thus has attracted considerable public attention worldwide in recent years (Chen et al. 2020; Şalcıoğlu et al. 2020). Numerous techniques have been developed to examine population genetic structure; among them, molecular genetic techniques have been widely used for their ability to identify and delineate cryptic stock structures where it may not be apparent from phenotypic or behavioural characteristics (Anderwald et al. 2011; Gonçalves da Silva et al. 2020). Such techniques have been widely applied in many marine species to examine their population structure for fisheries management purposes (e.g., Fernández et al. 2015; Pirog et al. 2019; Şalcıoğlu et al. 2020).

*Amphioctopusaegina* (Gray, 1849), commonly known as the marble or sand-bird octopus (Ignatius et al. 2011), is a medium-sized cephalopod species widely distributed in the coastal waters of the Northwest Pacific and Indian Ocean from India to China, as well as Malaysia and southern Indonesia (Dong 1988; Norman and Hochberg 2014). It contains high protein and low-fat content, as well as many mineral elements, and therefore has been the focal point of commercial fishing activities across its entire geographical range (Lei et al. 1956). In China, *A.aegina* dwells widely in coastal waters from south Fujian to Guangxi Province, and the annual fishery landings have reached hundreds of tons in some regions e.g., Hong Kong (Dong 1988). However, until now, there have been no population genetic studies investigating this species. There is only limited information available about the basic biology and ecology of this species (Ignatius et al. 2011; Promboon et al. 2011; Osman et al. 2014), which provides clues to its population genetic structure. For instance, the general life history of *A.aegina* is relatively short as its lifespan is less than one years in captivity (Ignatius et al. 2011). The mature females reproduce by laying small eggs and planktonic hatchlings throughout the year, primarily in January and October (Gibson et al. 2008; Prasopsook et al. 2022). Planktonic hatchings typically remain in a pelagic phase for one month before migrating to muddy substrates, their final habitat (Promboon et al. 2011; Iglesias et al. 2014). The reproductive strategy of producing small eggs and planktonic paralarvae may predict higher individual dispersal abilities compared to their counterparts and hence weaker genetic differentiation among populations (Gibson et al. 2008; Cahill et al. 2017). However, such inference is far from proven because, in addition to intrinsic factors such as like larval dispersal potential, population subdivision can also be largely influenced by extrinsic physical or ecological factors such as water depth, salinity and temperature that potentially act as a barrier to gene flow. Such ecological roles for population differentiation have been widely observed in many octopus species such as *Pareledoneturqueti* (Joubin, 1905), *P.charcoti* (Joubin, 1905), *Adelieledonepolymorpha* (Robson, 1930) (Strugnell et al. 2017), *Octopusmaya* (Voss et Solís-Ramírez, 1966) (Juárez et al. 2018) and *O.minor* (Sasaki, 1920) (Muhammad et al. 2019).

**Figure 1.**
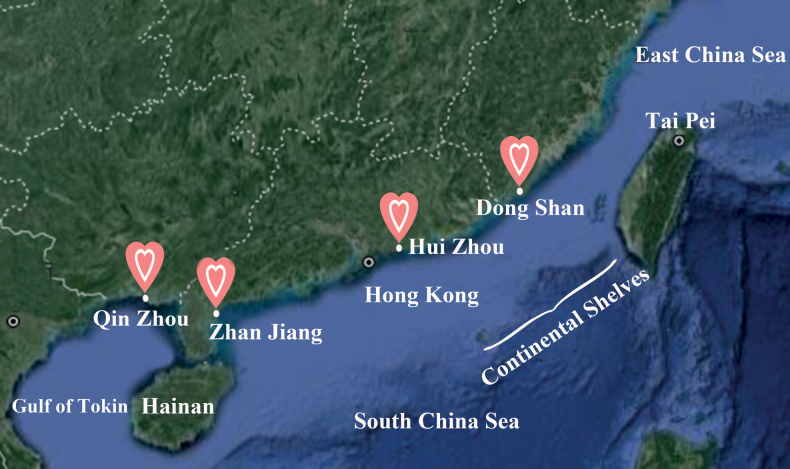
The sampling locations of *A.aegina* in waters of China collected from Dongshan, Huizhou, Zhanjiang and Qinzhou coastal waters.

In the present study, we sequenced the partial sequences of mitochondrial cytochrome b (cyt-b) DNA in *A.aegina* sampled from coastal waters of Dongshan (Fujian Province), Huizhou (Guangdong Province), Zhanjiang (Guangdong Province), and Qinzhou (Guangxi Province) throughout its distribution range (Fig. 1) to examine its population genetic structure. The outcomes of the present research would provide valuable information for scientific management and conservation of *A.aegina* resources in the coastal waters of China in the future.

## ﻿Materials and methods

### ﻿Sample collection and DNA extraction

We collected 74 specimens of adult *A.aegina*, from coastal waters of Dongshan, Huizhou, Zhanjiang and Qinzhou (Fig. 1, Table 1), from September 2017 to May 2019 through marine fishery surveys. Muscle tissue was taken from each individual and kept in 95% ethanol until the extraction of total genomic DNA using the standard phenol-chloroform method (Sambrook et al. 1989). All tissue sampling and DNA extraction processes complied with all relevant ethical regulations provided by the Institutional Animals Care and Use Committee of Zhejiang Ocean University.

**Table 1.** Sample location, sample size, and summary statistics of the genetic variation in each *A.aegina* population revealed by the mitochondrial cyt-b gene markers.

Sample locality	Code	Date	GPS Coordinates	Sample Size (No.)	Number of haplotypes (Hap)	Haplotype diversity (Hd)	Nucleotide diversity (pi)	Average number of differences (k)
Dongshan	DS	2017.7	23°41′42″N, 117°25′43″E	21	8	0.8048	0.0016	1.3429
Huizhou	HZ	2017.7	22°34′51″N, 114°31′31″E	13	4	0.4231	0.0008	0.6154
Zhanjiang	ZJ	2018.4	21°11′10″N, 110°25′2″E	18	3	0.7059	0.0012	0.9412
Qinzhou	QZ	2019.5	21°44′23″N, 108°35′53″E	22	1	0	0	0
Total				74	11	0.775	0.002	1.62

### ﻿Mitochondrial DNA amplification and sequencing

A 820-bp fragment of the mitochondrial cyt-b were used to examine genetic differentiation of all *A.aegina* populations. The amplifications were carried out using the primer combination (F 5’-CAAACTAACTACACCGCCTAA-3’; R 5’-GTTATTATTGTGAAGGTCCATT-3’) designed specifically for this locus of the octopuses according to the previous studies (Dou et al. 2020). The PCR analyses were conducted in a 25-µL volume containing 1 µL template DNA, 1× reaction buffer, 1.2 µL MgCl_2_, 2 µL dNTPs, 1 µL of each primer, and 0.1 µL Taq DNA polymerase (Promega, USA) using a PTC-200 (BIO-RAD, USA). Each reaction was performed under the following conditions: 5 min initial denaturation at 94 °C, 34 cycles of 30 s at 94 °C for denaturation, 45 s at 53 °C for annealing, and 1 min at 72 °C for extension, and a final extension at 72 °C for 5 min. All sets of PCRs included a negative control reaction in which all reagents were included, except for the template DNA. The amplification products were examined on a 1.5% agarose gel and stained with ethidium bromide. The Gel Extraction Mini Kit (Watson Bio Technologies, China) was used to purify the PCR products. Thereafter, the PCR products were visualized using 1.5% agarose gels and sent for sequencing to Sangon Inc., Shanghai, China. The obtained sequences were submitted to GenBank of the NCBI database and provided the accession numbers OM282997-OM283070.

### ﻿Data analysis

The sequences were edited and aligned using MEGA 6.0 software (Tamura et al. 2013). The molecular diversity parameters, such as the number of haplotypes, polymorphic sites, transitions, transversions, haplotype diversity (Hd), nucleotide diversity (pi) and mean number of pairwise differences (k) and their corresponding variances, were analyzed using DnaSP 5.10.01(Librado and Rozas 2009). The pairwise genetic distance was generated for phylogenetic tree reconstruction with MEGA 6.0 using the model of Kimura-2 parameters (Tamura et al. 2013). The phylogenetic analyses were performed using the Bayesian inference (BI) method implemented in MrBayes software (Huelsenbeck and Ronquist 2001). The best-fit substitution model was GTR+F+I, which was selected for BI analysis using ModelFinder based on Bayesian Information Criterion (Kalyaanamoorthy et al. 2017). The pairwise sequence divergences were also calculated between the lineages using DnaSP 5.10.01. In addition, genealogical relationships were examined by constructing haplotype networks using a reduced median network approach using PopART 1.7 software (Leigh and Bryant 2015). The population structure was measured with a hierarchical analysis of molecular variance (AMOVA) by incorporating the sequence divergence between haplotypes, and the significance of the covariance components was tested with 1000 permutations using ARLEQUIN v. 3.5 (Excoffier and Lischer 2010). In addition, the degrees of differentiation between the populations were evaluated with ΦST statistics. The significance of ΦST was tested by 10,000 permutations in ARLEQUIN v. 3.5. To evaluate the degree to which genetic differentiation of populations could be explained by isolation by distance (IBD), pairwise values of ΦST/(1-ΦST) were plotted against the geographical distance, which was measured as the shortest coastal distance between two sampling sites of *A.aegina*. Mantel tests were applied to determine the strength and significance of their co-relationship using reduced major axis (RMA) regression using IBDWS (Bohonak 2002). Uncertainties about appropriate nucleotide substitution rates for Cephalopoda mitochondrial cyt-b further confound the estimates of lineage divergence. Generally, a divergence rate of 2.0% per million years (My) has been calibrated for the mitogenome of invertebrate taxa (Shain et al. 2021), and 2.15–2.6% per My has been applied for the mitochondrial cyt-b for molluscan species (Sandoval-Castellanos et al. 2010). Using such nucleotide substitution rates for the mitochondrial cyt-b, the divergence time between the lineages was retro-calculated based on the strict clock (Ho and Duchêne 2014) method using BEAST v.2.6.7 (Bouckaert et al. 2014).

## ﻿Results

### ﻿Genetic variation in populations

We sequenced a 820 bp section of the cyt-b gene for 74 individuals from the four sample locations of this species. Sequence comparisons of the segment revealed 11 distinct haplotypes defined by 11 polymorphic sites, including six singleton and five parsimony-informative ones. The haplotype diversity (Hd) ranged from 0-0.8048, nucleotide diversity (pi) ranged from 0-0.0016, and the average number of nucleotide differences (k) ranged from 0–1.3429 for each population (see Table 1). The highest values of haplotype diversity, nucleotide diversity, and the mean number of pairwise differences were observed in the Dongshan population, while the lowest values were detected in the Qinzhou population, in which only one haplotype and no nucleotide variation were identified.

### ﻿Population genetic structure and phylogeography

**Figure 2.**
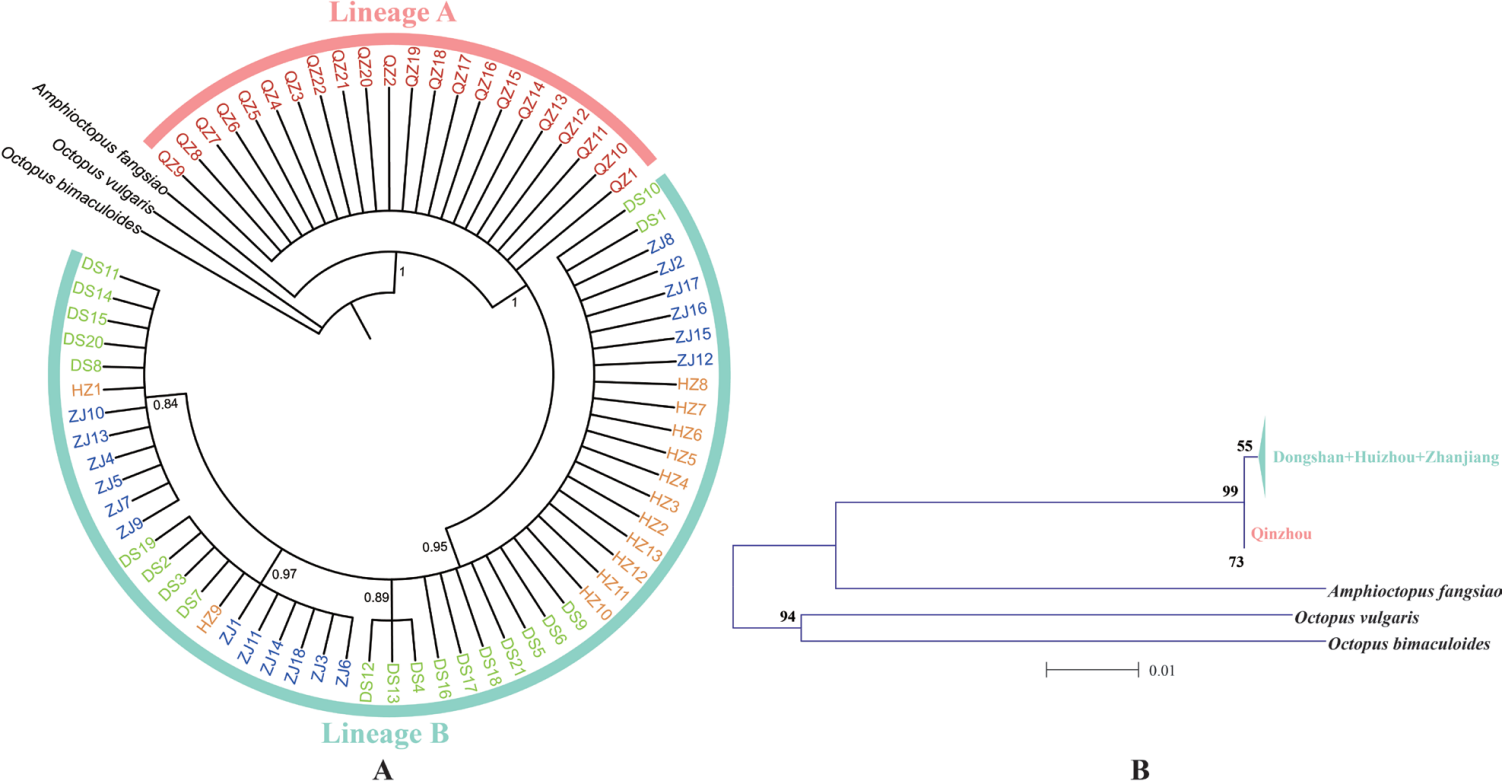
The phylogenetic tree constructed from the mitochondrial cytochrome b DNA sequences **A** the BI tree constructed from all individuals; Two lineages (lineage A and B) were revealed in the constructed tree **B** the NJ tree constructed from the four populations. Posterior probabilities of > 0.80 were shown in A. *Amphioctopusfangsiao*, *Octopusbimaculoides*, and *O.vulguris* were used as the outgroups when the phylogenetic trees were constructed.

**Figure 3.**
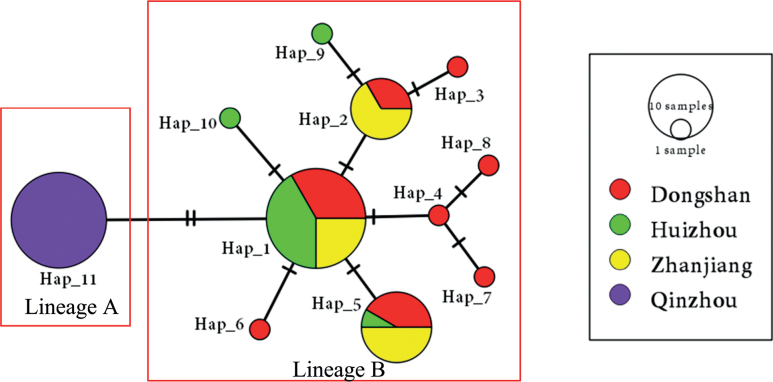
The median-joining network constructed from the haplotypes of the mitochondrial cytochrome b DNA sequences. Different colors represent the different populations analyzed. The size of the circle in the network is proportional to the haplotype frequency observed in populations. The lineages A and B are marked with red boxes.

The BI trees constructed from the mitochondrial cyt-b DNA sequences revealed two reciprocal monophyletic lineages with comparatively high posterior probabilities (≥ 0.84) (Fig. 2). In lineage A, sequences from the Qinzhou population clustered together, and while in lineage B, the sequences from the other three populations (Dongshan, Huizhou and Zhanjiang) clustered together. Following the pattern of the Bayesian tree, the median-joining network based on the mitochondrial cyt-b loci also separated the two lineages corresponding to Qinzhou and the other three populations (Fig. 3). The three populations shared haplotypes while the Qinzhou population had its own haplotype, and there were no shared haplotypes; two fixed nucleotide differentiations have occurred in haplotypes between the two lineages (Fig. 3). Such differentiation between the lineages A and B was also supported by AMOVA analyses because 69.75% of the total genetic variation was detected between the two lineages while only 28.84% and 1.41% of the total genetic variation were detected within population and among populations within lineages (see Table 2). Pairwise ΦST values among populations between lineages were high (0.7090–0.8130) and significant (p < 0.01) after sequential Bonferroni correction, also suggesting their obvious differentiation and the limited gene flow (Nm, 0.1150–0.2052) between the observed two lineages (see Table 3).

**Table 2.** Molecular variance within and among *A.aegina* populations as revealed by the mitochondrial cyt-b DNA sequences.

Source of variation	df	Sum of squares	Variance component	Percentage (%)
Among lineages	1	32.279	0.9859 Va	69.75
Among population with in lineages	2	1.495	0.0200 Vb	1.41
Within population	71	28.947	0.4077 Vc	28.84
Total	74	62.72	1.4135	
Fixation Index	0.7116			

**Table 3.** Pairwise Φst and Nm values among four populations of *A.aegina* as revealed by the mitochondrial cyt-b DNA sequences. Note: Φst and Nm values are given below and above the diagonal, respectively; ** represents a *P* value < 0.01.

Populations	Dongshan	Huizhou	Zhanjiang	Qinzhou
Dongshan	–	37.9615	499.5	0.2052
Huizhou	0.013	–	4.5	0.115
Zhanjiang	0.001	0.1	–	0.1435
Qinzhou	0.7090**	0.8130**	0.7770**	–

### ﻿IBD test and divergence time estimated

**Figure 4.**
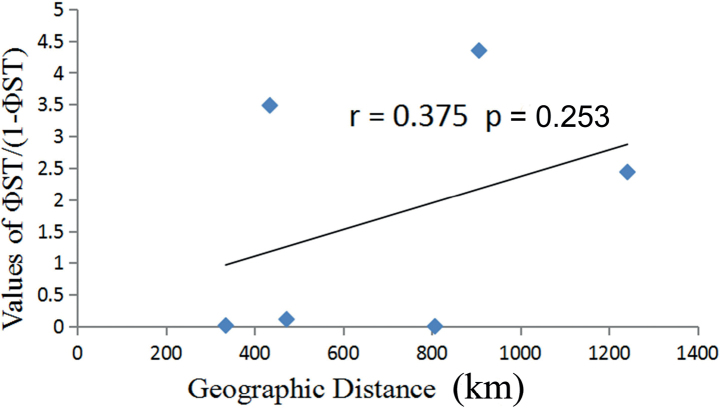
The plot of the pairwise estimates of ΦST/(1-ΦST) and the geographic distance between populations of *A.aegina*. The x and y axis represent the geographic distances and the values of ΦST/(1-ΦST) among populations, respectively.

**Figure 5.**
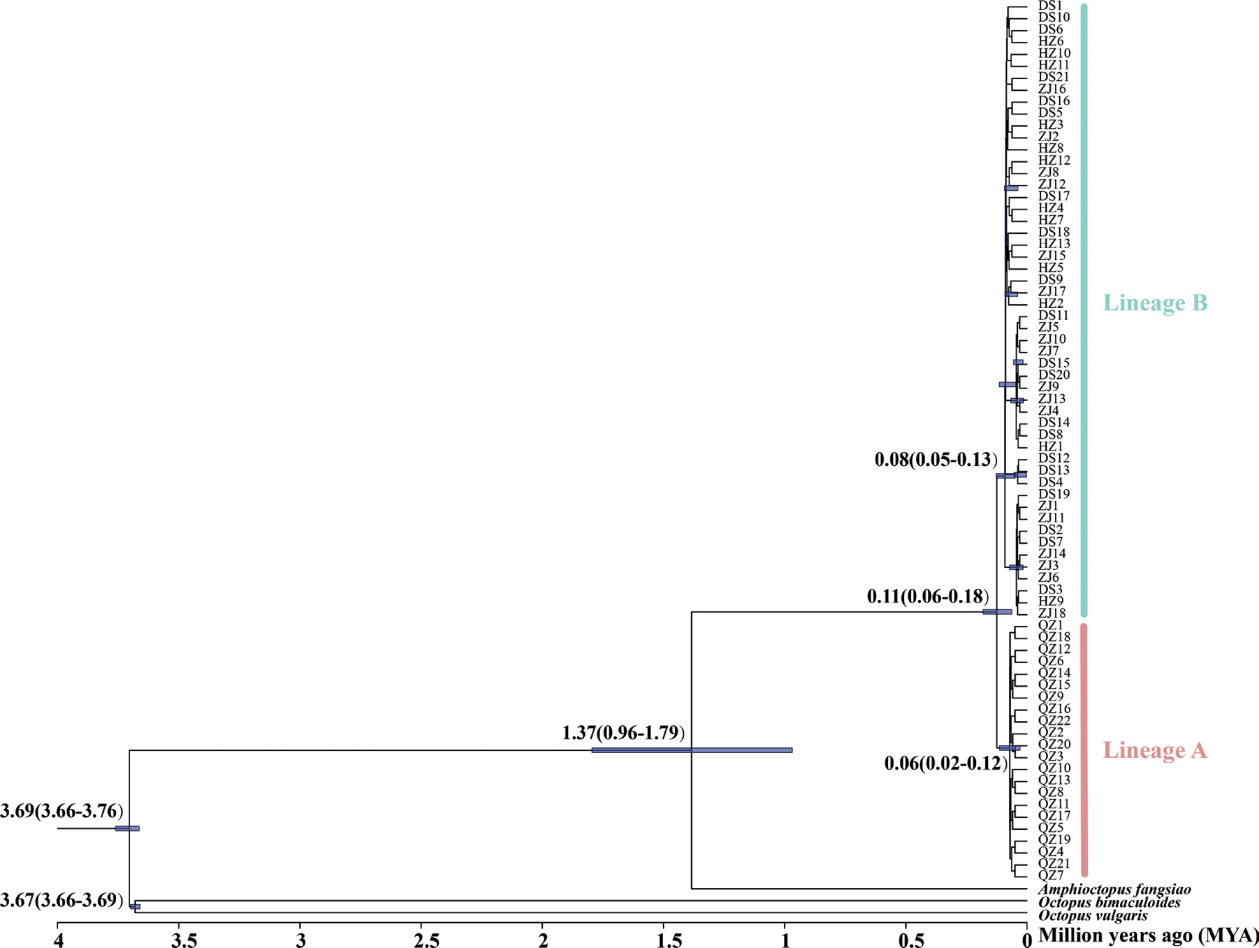
Chronogram with divergence times for *A.aegina* lineages generated by BEAST. Blue bars on the nodes represent 95% highest posterior density for divergence times. The scale indicates time in millions of years (Mya).

Pairwise values of ΦST/(1-ΦST) among the four populations were plotted against the geographical distances (Fig. 4), and Mantel’s test indicated that the correlation between pairwise differentiation and geographical distances was low and not significant (r = 0.375, p = 0.253), which may suggest that there are contemporary environmental factors shaping the genetic structure of *A.aegina*. The largest genetic distances were observed between Qinzhou and the other three populations, and the pairwise genetic distance between the two lineages was 0.0032. Using the molecular clock of 2.15–2.60% per million years usually applied for the mitochondrial cyt-b for molluscan species, the lineage A would have diverged from the lineage B about 110 ka BP (95% HPD, 60–180 ka BP) (Fig. 5).

## ﻿Discussion

In the present study, we examined the genetic diversity and the population genetic structure in four populations of *A.aegina* sampled throughout its full distribution range in China using the mitochondrial cyt-b DNA sequences. The results show that *A.aegina* populations in China generally possess low (Qinzhou) to high (Dongshan) genetic diversity, which is usually observed in cephalopods (Brierley et al. 1993; Shaw et al. 1999). The Dongshan population usually has higher genetic diversity compared to the other three populations. A higher genetic diversity in the Dongshan population may predict their greater adaptive potential when faced with environmental changes and higher priorities for conservation as an evolutionarily significant unit (Harrisson et al. 2016).

However, contrasting to our earlier inference (Introduction) of low differentiation among populations due to their reproductive strategy of producing small eggs and planktonic paralarvae which usually leads to high larval dispersal, and hence high gene flow (Gibson et al. 2008), a significant population structure has been revealed in *A.aegina* using the mitochondrial cyt-b DNA sequences. The BI tree constructed from these sequences indicates two reciprocal monophyletic lineages (Fig. 2), which correspond to the Qinzhou population and the other three populations of the Dongshan, Huizhou, and Zhanjiang. Such divergence between the two lineages is further supported by the median-joining network of haplotypes (Fig. 3), the pairwise ΦST values between populations (Table 3), and the results of the AMOVA analyses (Table 2). The genetic structure between the two lineages was deep and significant with a ΦST value of 0.7116 (p < 0.01), which has caused low gene flow (Nm 0.2026). Such high ΦST values are unusual in marine populations since lineage divergence would be considered moderate when the ΦST value reaches 0.05, while a ΦST value greater than 0.25 indicate very high genetic differentiation between populations. The high ΦST values may arise mainly from the two fixed nucleotides occurring in haplotypes between the two lineages (Fig. 3), which indicate a recent divergence. Such inference is also supported by the generally low genetic distance (0.0032) between them, which falls into a scope of intraspecific-phylogroup divergence because intraspecific-phylogroup are typically distinguished consistently by 0~0.2–0.6% sequence divergence for mtDNA(Avise and Walker 1999). More specifically for cyt-b, (Bradley and Baker 2001) claimed that values < 2% would equal intra-specific variation, while values between 2 and 11% would merit additional study, and values > 11–13% would be indicative of specific recognition. The recent lineage differentiation was further supported by the divergence time calibrated between the two lineages, which falls into a time scope of 60–180 (mean 110) ka BP.

Many factors may potentially contribute to the genetic differentiation among marine populations such as geographic isolation, the influence of ocean currents, life history features, and ancient geological events (Gao et al. 2016). To evaluate the degree to which genetic differentiation of populations could be explained by geographic isolation, Mantel tests were conducted to determine the significance between genetic and geographical distances and the results indicated no significant correlation with geographical distance (r = 0.375, p = 0.253). These results may suggest that some other contemporary environmental factors may play important roles in shaping the genetic structure of *A.aegina*. From the sampling localities of the specimens, Dongshan and Zhanjiang (807.25 km apart) and Huizhou and Zhanjiang (472.25 km apart) are much farther apart than Qinzhou and Zhanjiang (424.44 km). However, the corresponding genetic differences among these populations show an opposite trend as revealed by pairwise ΦST values (Table 3). This suggests that Leizhou Peninsula and the adjacent Hainan Island between Zhanjiang and Qinzhou may act as a geographical barrier to gene flow in *A.aegina* populations. The Leizhou Peninsula stands as the third largest peninsula in China, while paired with Hainan Island, the second largest island in the country. Together, they create a natural geographic barrier between the Gulf of Tonkin and the remaining South China Sea, allowing only the passage of the slender and elongated Qinzhou Strait (Zhao et al. 2009; Sun and Tang 2018). Such physical isolation also represents barriers to gene flow for many marine species and significant genetic differentiation has been detected in populations dwelling on the two sides of the peninsula (Zhao et al. 2009; Sun and Tang 2018; Zhao et al. 2018). For this reason (Spalding et al. 2007) even recognized the regions on the two sides of the Leizhou Peninsula as two different marine ecoregions: the Gulf of Tonkin and Southern China ecoregions. From the diverged time calibrated for the two lineages (Fig. 5), 110 ka BP roughly falls into a time scale of the last glacial period (~ 10–120 ka BP) (Rasmussen et al. 2014), during which the Gulf of Tonkin became isolated by the Leizhou Peninsula due to the lower sea level (Lambeck et al. 2002). Such a molecular-clock estimates may suggest a differentiation by the last glacial period isolation of the Leizhou Peninsula in *A.aegina* populations, as had already been indicated in several other marine taxa in this region (Zhao et al. 2009; Sun and Tang 2018; Zhao et al. 2018).

The observed genetic diversity in *A.aegina*, which ranges from low to moderate levels, highlights the urgent need for conservation measures to be implemented for this significant species in the fishing industry. The recent but significant genetic break existing among populations prove the necessity for regarding Qinzhou and the remaining three populations as the significant separate evolutionarily units for future conservation and management.

## ﻿Additional information

### Author ORCIDs

Amna Sulaman https://orcid.org/0000-0002-5761-5503

### Data availability

All of the data that support the findings of this study are available in the main text.

## References

[B1] Anderwald P Daníelsdóttir AK Haug T Larsen F Lesage V Reid RJ Víkingsson GA Hoelzel AR (2011) Possible cryptic stock structure for minke whales in the North Atlantic: Implications for conservation and management. Biological Conservation 144(10): 2479–2489. 10.1016/j.biocon.2011.07.002

[B2] Avise JC Walker D (1999) Species realities and numbers in sexual vertebrates: Perspectives from an asexually transmitted genome. Proceedings of the National Academy of Sciences of the United States of America 96(3): 992–995. 10.1073/pnas.96.3.992 9927681 PMC15338

[B3] Bohonak A (2002) IBD (isolation by distance): A program for analyses of isolation by distance. The Journal of Heredity 93(2): 153–154. 10.1093/jhered/93.2.153 12140277

[B4] Bouckaert R Heled J Kühnert D Vaughan T Wu C-H Xie D Suchard MA Rambaut A Drummond AJ (2014) BEAST 2: A software platform for Bayesian evolutionary analysis. PLoS Computational Biology 10(4): e1003537. 10.1371/journal.pcbi.1003537 PMC3985171 24722319

[B5] Bradley RD Baker RJ (2001) A test of the genetic species concept: Cytochrome-b sequences and mammals. Journal of Mammalogy 82(4): 960–973. 10.1644/1545-1542(2001)082<0960:ATOTGS>2.0.CO;2 PMC2771874 19890476

[B6] Brierley A Rodhouse P Thorpe J Clarke M (1993) Genetic evidence of population heterogeneity and cryptic speciation in the ommastrephid squid *Martialiahyadesi* from the Patagonian Shelf and Antarctic Polar Frontal Zone. Marine Biology 116(4): 593–602. 10.1007/BF00355478

[B7] Cahill AE De Jode A Dubois S Bouzaza Z Aurelle D Boissin E Chabrol O David R Egea E Ledoux JB Mérigot B Weber AA-T Chenuil A (2017) A multispecies approach reveals hot spots and cold spots of diversity and connectivity in invertebrate species with contrasting dispersal modes. Molecular Ecology 26(23): 6563–6577. 10.1111/mec.14389 29087018

[B8] Chen W Li C Chen F Li Y Yang J Li J Li X (2020) Phylogeographic analyses of a migratory freshwater fish (*Megalobramaterminalis*) reveal a shallow genetic structure and pronounced effects of sea-level changes. Gene 737: е144478. 10.1016/j.gene.2020.144478 32061762

[B9] Dong Z (1988) Fauna Sinica, Phylum Mollusca, Class Cephalopoda. Science Press, Beijing.

[B10] Dou C Muhammad F Liu L Gong L Chen Y Guo B Lü Z (2020) Population diversity of *Cistopusindicus* inferred from mitochondrial DNA (Cytochrome b) variation from China and Vietnam. Molluscan Research 40(1): 1–7. 10.1080/13235818.2019.1688635

[B11] Excoffier L Lischer HE (2010) Arlequin suite ver 3.5: A new series of programs to perform population genetics analyses under Linux and Windows. Molecular Ecology Resources 10(3): 564–567. 10.1111/j.1755-0998.2010.02847.x 21565059

[B12] Fernández R Lemer S McIntyre E Giribet G (2015) Comparative phylogeography and population genetic structure of three widespread mollusc species in the Mediterranean and near Atlantic. Marine Ecology 36(3): 701–715. 10.1111/maec.12178

[B13] Gao X Zheng X Bo Q Li Q (2016) Population genetics of the common long-armed octopus *Octopusminor* (Sasaki, 1920) (Cephalopoda: Octopoda) in Chinese waters based on microsatellite analysis. Biochemical Systematics and Ecology 66: 129–136. 10.1016/j.bse.2016.03.014

[B14] Garoia F Guarniero I Grifoni D Marzola S Tinti F (2007) Comparative analysis of AFLPs and SSRs efficiency in resolving population genetic structure of Mediterranean *Soleavulgaris*. Molecular Ecology 16(7): 1377–1387. 10.1111/j.1365-294X.2007.03247.x 17391263

[B15] Gibson R Atkinson R Gordon J (2008) Biology of the planktonic stages of benthic octopuses. Oceanography and Marine Biology – an Annual Review 46: 105–106. 10.1201/9781420065756-6

[B16] Gonçalves da Silva A Barendse W Kijas J England PR Hoelzel AR (2020) Genomic data suggest environmental drivers of fish population structure in the deep sea: A case study for the orange roughy (*Hoplostethusatlanticus*). Journal of Applied Ecology 57(2): 296–306. 10.1111/1365-2664.13534

[B17] Harrisson KA Pavlova A Gonçalves da Silva A Rose R Bull JK Lancaster ML Murray N Quin B Menkhorst P Magrath MJ Sunnucks P (2016) Scope for genetic rescue of an endangered subspecies though re‐establishing natural gene flow with another subspecies. Molecular Ecology 25(6): 1242–1258. 10.1111/mec.13547 26820991

[B18] Ho SY Duchêne S (2014) Molecular‐clock methods for estimating evolutionary rates and timescales. Molecular Ecology 23(24): 5947–5965. 10.1111/mec.12953 25290107

[B19] Huelsenbeck JP Ronquist F (2001) MRBAYES: Bayesian inference of phylogenetic trees. Bioinformatics 17(8): 754–755. 10.1093/bioinformatics/17.8.754 11524383

[B20] Iglesias J Fuentes L Villanueva R (2014) Cephalopod culture. Springer Science & Business Media. 10.1007/978-94-017-8648-5

[B21] Ignatius B Srinivasan M Balakrishnan S (2011) Reproductive traits of sandbird octopus, *Amphioctopusaegina* (Gray, 1849) from Mandapam coastal waters (Palk Bay), Southeast Coast of India. Ocean Science Journal 46(3): 145–154. 10.1007/s12601-011-0013-z

[B22] Juárez OE Enríquez L Camarena-Rosales F Arena L Galindo-Sánchez CE Lafarga-De la Cruz F López-Galindo L Nambo K Rosas C (2018) Genetic monitoring of the Mexican four-eyed octopus *Octopusmaya* population: New insights and perspectives for the fishery management. Fisheries Research 206: 109–114. 10.1016/j.fishres.2018.05.002

[B23] Kalyaanamoorthy S Minh BQ Wong TK Von Haeseler A Jermiin LS (2017) ModelFinder: Fast model selection for accurate phylogenetic estimates. Nature Methods 14(6): 587–589. 10.1038/nmeth.4285 28481363 PMC5453245

[B24] Lambeck K Esat TM Potter E-K (2002) Links between climate and sea levels for the past three million years. Nature 419(6903): 199–206. 10.1038/nature01089 12226674

[B25] Lei X Zhao S Yang Z Fan X WU H (1956) The nutrient analysis and evaluation of *Octopusdollfusi* in South China Sea. Acta Nutrimenta Sinica.

[B26] Leigh JW Bryant D (2015) POPART: Full-feature software for haplotype network construction. Methods in Ecology and Evolution 6(9): 1110–1116. 10.1111/2041-210X.12410

[B27] Librado P Rozas J (2009) DnaSP v5: A software for comprehensive analysis of DNA polymorphism data. Bioinformatics 25(11): 1451–1452. 10.1093/bioinformatics/btp187 19346325

[B28] Muhammad F Chen W Liu L Gong L Du X Shafi M Lü Z (2019) Genetic structure of *Amphioctopusfangsiao* (Mollusca, Cephalopoda) in Chinese waters inferred from variation in three mtDNA genes (ATPase 6, ND2, and ND5). Hydrobiologia 838(1): 111–119. 10.1007/s10750-019-03981-9

[B29] Norman M Hochberg F (2014) Octopods and vampire squids.

[B30] Osman IH Gabr HR El-Etreby SG Mohammed SZ (2014) Morphometric variations and genetic analysis of Lessepsian migrant *Octopusaegina* (Cephalopoda: Octopodidae). Journal of King Abdulaziz University 25: 1–23. 10.4197/Mar.25-2.2

[B31] Pirog A Jaquemet S Ravigné V Cliff G Clua E Holmes BJ Hussey NE Nevill JE Temple AJ Berggren P Vigliola L Magalon H (2019) Genetic population structure and demography of an apex predator, the tiger shark *Galeocerdocuvier*. Ecology and Evolution 9(10): 5551–5571. 10.1002/ece3.5111 31160982 PMC6540675

[B32] Prasopsook P Sukhsangchan C Whanphetch N (2022) Embryonic development and external morphology of *Amphioctopusaegina* (Gray, 1849) (Cephalopoda: Octopodidae) in Thailand. ScienceAsia 48(4): 1–48. 10.2306/scienceasia1513-1874.2022.057

[B33] Promboon P Nabhitabhata J Duengdee T (2011) Life cycle of the marbled octopus, *Amphioctopusaegina* (Gray) (Cephalopoda: Octopodidae) reared in the laboratory. Scientia Marina 75(4): 811–821. 10.3989/scimar.2011.75n4811

[B34] Rasmussen SO Bigler M Blockley SP Blunier T Buchardt SL Clausen HB Cvijanovic I Dahl-Jensen D Johnsen SJ Fischer H Gkinis V Guillevic M Hoek WZ Lowe JJ Pedro JB Popp T Seierstad IK Steffensen JP Svensson AM Vallelonga P Vinther BM Walker MJC Wheatley JJ Winstrup M (2014) A stratigraphic framework for abrupt climatic changes during the Last Glacial period based on three synchronized Greenland ice-core records: Refining and extending the INTIMATE event stratigraphy. Quaternary Science Reviews 106: 14–28. 10.1016/j.quascirev.2014.09.007

[B35] Şalcıoğlu A Gubili C Krey G Sönmez AY Bilgin R (2020) Phylogeography and population dynamics of the Eastern Mediterranean whiting (*Merlangiusmerlangus*) from the Black Sea, the Turkish Straits System, and the North Aegean Sea. Fisheries Research 229: е105614. 10.1016/j.fishres.2020.105614

[B36] Sambrook J Fritsch EF Maniatis T (1989) Molecular cloning: a laboratory manual. Cold Spring Harbor Laboratory Press.

[B37] Sandoval-Castellanos E Uribe-Alcocer M Díaz-Jaimes P (2010) Population genetic structure of the Humboldt squid (*Dosidicusgigas* d’Orbigny, 1835) inferred by mitochondrial DNA analysis. Journal of Experimental Marine Biology Ecology Evolution 385(1–2): 73–78. 10.1016/j.jembe.2009.12.015

[B38] Shain DH Novis PM Cridge AG Zawierucha K Geneva AJ Dearden PK (2021) Five animal phyla in glacier ice reveal unprecedented biodiversity in New Zealand’s southern Alps. Scientific Reports 11(1): е3898. 10.1038/s41598-021-83256-3 PMC7887191 33594128

[B39] Shaw P Pierce G Boyle P (1999) Subtle population structuring within a highly vagile marine invertebrate, the veined squid *Loligoforbesi*, demonstrated with microsatellite DNA markers. Molecular Ecology 8(3): 407–417. 10.1046/j.1365-294X.1999.00588.x

[B40] Spalding MD Fox HE Allen GR Davidson N Ferdaña ZA Finlayson M Halpern BS Jorge MA Lombana A Lourie SA Martin KD McManus E Molnar J Recchia CA Robertson J (2007) Marine ecoregions of the world: A bioregionalization of coastal and shelf areas. Bioscience 57(7): 573–583. 10.1641/B570707

[B41] Strugnell JM Allcock AL Watts PC (2017) Closely related octopus species show different spatial genetic structures in response to the Antarctic seascape. Ecology and Evolution 7(19): 8087–8099. 10.1002/ece3.3327 29043058 PMC5632630

[B42] Sun P Tang B (2018) Low mtDNA variation and shallow population structure of the Chinese pomfret *Pampuschinensis* along the China coast. Journal of Fish Biology 92(1): 214–228. 10.1111/jfb.13515 29205347

[B43] Tamura K Stecher G Peterson D Filipski A Kumar S (2013) MEGA6: Molecular evolutionary genetics analysis version 6.0. Molecular Biology and Evolution 30(12): 2725–2729. 10.1093/molbev/mst197 24132122 PMC3840312

[B44] Zhao Y Li Q Kong L Mao Y (2009) Genetic and morphological variation in the venus clam *Cyclinasinensis* along the coast of China. Hydrobiologia 635(1): 227–235. 10.1007/s10750-009-9916-4

[B45] Zhao Y Peng W Guo H Chen B Zhou Z Xu J Zhang D Xu P (2018) Population genomics reveals genetic divergence and adaptive differentiation of Chinese sea bass (*Lateolabraxmaculatus*). Marine Biotechnology 20(1): 45–59. 10.1007/s10126-017-9786-0 29256104

